# Human CD8^+^ T-cells Recognizing Peptides from *Mycobacterium tuberculosis* (*Mtb*) Presented by HLA-E Have an Unorthodox Th2-like, Multifunctional, *Mtb* Inhibitory Phenotype and Represent a Novel Human T-cell Subset

**DOI:** 10.1371/journal.ppat.1004671

**Published:** 2015-03-24

**Authors:** Krista E. van Meijgaarden, Mariëlle C. Haks, Nadia Caccamo, Francesco Dieli, Tom H. M. Ottenhoff, Simone A. Joosten

**Affiliations:** 1 Department of Infectious Diseases, Leiden University Medical Center, Leiden, The Netherlands; 2 Central Laboratory for Advanced Diagnostic and Biomedical Research (CLADIBIOR), Dipartimento di Biopatologia e Biotecnologie Mediche e Forensi, Università di Palermo, Palermo, Italy; Weill Medical College of Cornell University, UNITED STATES

## Abstract

Mycobacterial antigens are not exclusively presented to T-cells by classical HLA-class Ia and HLA-class II molecules, but also through alternative antigen presentation molecules such as CD1a/b/c, MR1 and HLA-E. We recently described mycobacterial peptides that are presented in HLA-E and recognized by CD8^+^ T-cells. Using T-cell cloning, phenotyping, microbiological, functional and RNA-expression analyses, we report here that these T-cells can exert cytolytic or suppressive functions, inhibit mycobacterial growth, yet express GATA3, produce Th2 cytokines (IL-4,-5,-10,-13) and activate B-cells via IL-4. In TB patients, Mtb specific cells were detectable by peptide-HLA-E tetramers, and IL-4 and IL-13 were produced following peptide stimulation. These results identify a novel human T-cell subset with an unorthodox, multifunctional Th2 like phenotype and cytolytic or regulatory capacities, which is involved in the human immune response to mycobacteria and demonstrable in active TB patients’ blood. The results challenge the current dogma that only Th1 cells are able to inhibit Mtb growth and clearly show that Th2 like cells can strongly inhibit outgrowth of Mtb from human macrophages. These insights significantly expand our understanding of the immune response in infectious disease.

## Author Summary

Pathogens like *Mycobacterium tuberculosis* (Mtb) are recognized by human T-cells following their presentation in HLA molecules. HLA class I molecules can be divided into two types, classical as well as non-classical HLA molecules. Here we studied the non-classical HLA family member, HLA-E, which displays only minimal genetic variation between individuals and is relative resistant to down modulation by HIV infection. We have characterized the T-cells that recognize Mtb in the context of HLA-E in detail and found that these human CD8^+^ T-cells had unexpected, unorthodox properties: in contrast to most classical CD8^+^ T-cells, the T-cells activated by HLA-E uniquely produced Th2 (IL-4, IL-5, IL-13) instead of the usual Th1 cytokines, and were able to activate B-cells and induced cytokine production by these B-cells. Moreover, these HLA-E restricted CD8^+^ T-cells inhibited Mtb growth inside cells, an important property to contribute to resolution of the infection. Thus these T-cells represent a new player in the human immune response to infection, and add B-cell activation to the key pathways following infection with Mtb.

## Introduction

Tuberculosis (TB) remains a major global threat because current interventions are unable to prevent or treat infection adequately. *Mycobacterium tuberculosis* (Mtb) is an intracellular pathogen that has evolved a myriad of effective evasion strategies to thwart host defence mechanisms. Due to increasing drug resistance, the continued impact of HIV co-infections and, more recently, the increasing impact of non-infectious co-morbidities in TB endemic areas, in particular obesity- associated type II diabetes mellitus, TB is unlikely to be conquered any time soon [1–5]. A major obstacle in designing more effective vaccination strategies against TB is our incomplete understanding of the human host response to Mtb, in particular the determinants that control protective immunity versus disease susceptibility [1–4]. This is e.g. illustrated by the unexpected failure of a recent vaccine trial using MVA85A, which was designed to boost BCG primed CD4^+^ Th1 cell responses, considered to be key to protection [6]. These results have led to a wide re-evaluation of current paradigms of the human immune response and protective host defence in TB, including the identification of major knowledge gaps.

Current efforts to develop better TB vaccines include the development of subunit as well as live mycobacterial vaccines, and have generally aimed at inducing classical HLA class II and Ia restricted CD4 and CD8 Th1 cells. While canonical HLA class Ia and class II molecules are highly polymorphic, the HLA class Ib family contains only few allelic variants: 2, 4 and 10 for HLA-E, -F and G, respectively [7]. Recently a novel coding variant for HLA-E was described, but this variation is unlikely to involve alternative peptide binding [8]. All described amino acid variations in HLA-E are located distant from the peptide binding groove, and in agreement with this, no differences in peptide binding capacities have been observed [9]. Physiologically, HLA-E is an interesting candidate antigen presentation molecule for new TB vaccine antigens. HLA-E is almost monomorphic, and its expression is enriched on Mtb phagosomes compared to classical class Ia family members, facilitating HLA-E peptide loading in Mtb infected cells [10]. Moreover, Mtb infected airway epithelial cells can also present Mtb antigens in HLA-E [11]. In addition, due to a mutation in the intracellular domain HLA class Ib family members are not sensitive to downregulation by HIV-nef proteins and thus should remain capable of presenting mycobacterial antigens during concomitant HIV-TB infection.

Qa-1, the murine equivalent of human HLA-E, is functionally important in mouse models of (intracellular) infectious diseases, underlining the functional contribution of non-classical class Ib restricted CD8^+^ T-cells to host defense. Pathogen specific Qa-1 restricted CD8^+^ T-cells can lyse infected target cells efficiently [12,13]. Antigens recognized from *Salmonella typhimurium* mimicked murine heat-shock proteins, resulting in potential recognition of stressed cells [12,14]. Antigen processing defects have been indicated as important triggers of Qa-1 restricted CTLs, supporting their role in immune-surveillance [13–15]. However, Qa-1-restricted CD8^+^ T-cells can also have regulatory activity, and can hamper efficient viral clearance in murine LCMV infection [16]. Qa-1 knockout have enhanced antiviral responses, resulting in reduced inflammation both in acute and chronic phases of the infection [16].

In humans, HLA-E restricted responses have been associated with effector responses towards infectious pathogens: HLA-E restricted responses were observed against *cytomegalovirus* (CMV)[17–19], *Salmonella typhi* [20,21], Mtb [22,23] and *Epstein-Barr virus* (EBV)[24]. Recognition of CMV and *Salmonella typhi* in the context of HLA-E resulted in production of IFNγ, as well as lysis of infected target cells by granule-dependent pathways [18,20]. HLA-E restricted responses to *Salmonella typhi* persisted long-term as they were detectable up to 2 years post-vaccination [21]. We have previously described the first peptides derived from Mtb that can be presented by HLA-E to human CD8^+^ T-cells. We demonstrated that some of these polyclonal CD8^+^ T-cells could suppress proliferation and cytokine production of Th1 cells, thus representing a subset of human CD8^+^ regulatory T-cells (Tregs), whereas another subset of CD8^+^ T-cells had cytolytic activity towards BCG infected monocytes [23].

To unravel the function and specificity of human HLA-E restricted Mtb reactive CD8^+^ T-cells in more detail at the single cell level, we have performed an in depth analysis of CD8^+^ T-cell clones that recognize selected HLA-E presented Mtb peptides. While most Mtb reactive T-cells described have Th1 like functions, by contrast we find that HLA-E restricted Mtb reactive CD8^+^ T cell clones have unorthodox phenotypes, with many characteristics of Th2 cells, including the expression of the type 2 associated transcription factor GATA3, type 2 cytokine expression, capacity to activate B-cells, and either suppressive or cytolytic functions. Importantly, these cells are present in patients with TB. Moreover, they are able to inhibit intracellular growth of Mtb, challenging the dogma that Th1 but not Th2 cells can contribute to mycobacterial growth control. These non-classical T-cells thus represent an important and novel ‘multifunctional’ subset engaged in the human immune response to infection.

## Results

### T-cell receptor mediated recognition of Mtb peptides presented by HLA-E

Mtb derived peptides can be presented to CD8^+^ T-cells by the non-classical HLA class Ib molecule HLA-E [23]. To study this arm of the human T-cell response in more depth, we generated T-cell clones specific for either one of 2 Mtb derived peptides that are presented by HLA-E, using limiting dilution cultures. Thirty CD8^+^ T-cell clones from 2 independent donors were obtained, 16 of which could be characterized in more detail: 10 from donor 2 reactive against peptide #62 (derived from Rv2997, alanine rich dehydrogenase involved in secondary metabolites biosynthesis, transport and catabolism), and 6 from donor 6 reactive against peptide #68 (derived from Rv1523, methyltransferase involved in secondary metabolites biosynthesis, transport and catabolism). In all experiments the majority of these clones was included, guided by cell number availability. Each experiment included at a minimum 5 clones from each donor. All assays were performed on at least 12 independent T-cell clones in at least 3 independent experiments. The total set of combined data for all individual clones is given in S1 Table.

Phenotyping by flow cytometry showed that 15 of the 16 clones had a CD3^+^CD8^+^ and 1/16 a CD3^+^CD4^+^CD8^+^ double positive phenotype (Table 1, S1 Table). The gating criteria are shown in S1 Fig. compliant with MIATA guidelines [25], gate settings were determined using fresh PBMCs (S1A Fig.) and applied to our T-cell clones (S1B Fig.). Twelve out of 13 T-cell clones tested expressed the αβ T-cell receptor (TCR), whereas 1 clone expressed the γδ TCR (Table 1). Moreover, while CD56 expression was observed in 1/13 clones, all clones lacked CD94, NKG2A, NKG2B and NKG2D, potential ligands for HLA-E (Table 1, S1B Fig.).

**Table 1:** Phenotype of human CD8^+^ T-cell clones reactive with Mtb peptides presented by HLA-E.

marker	# clones	criterion for positivity
T cell subset markers	-	-
CD3+	**16/16**	>90%
CD4+	0/16	>90%
CD8+	**15/16**	>90%
CD4+8+	1/16	>90%
TCRαβ	**12/13**	>75%
TCRγδ	1/13	>75%
CD45RA-CCR7+	**12/13**	>70%
CD45RA-CCR7-	1/13	>70%
CCR7+	**12/13**	>70%
CCR7+CD27-	**11/13**	>70%
NK markers	-	-
CD16+	0/13	>50%
CD56+	1/13	>50%
CD94+	0/13	>10%
NKG2A+	0/13	>10%
NKG2C+	0/13	>10%
NKG2D+	0/13	>10%
Treg markers	-	-
CD25+	**16/16**	>50%
CD39+	**11/16**	>50%
LAG3+	**13/16**	>50%
CD127+	0/13	>50%
CTLA4+	**13/13**	>50%
Markers of cytotoxicity	-	-
GranzymeA+	**13/13**	>50%
GranzymeB+	**11/13**	>50%
Granulysin+	**8/13**	>50%
Perforin+	3/13	>50%
Transcription factors	-	-
Tbet+	0/13	>10%
RORC+	0/13	>10%
GATA3+	**13/13**	>10%
eomes+	**9/13**	>10%
FoxP3+	0/13	>10%

Phenotype of CD8^+^ T-cell clones was determined using flow cytometry for all 16 individual clones, and results were scored for all clones tested for the specific marker (combination) according to the percentage of positive cells as indicated in the last column, the cut-off was set based on population characteristics and staining patterns on PBMC. Individual data (% of cells expressing particular markers) for all clones can be found in S1 Table.

**Fig 1:**
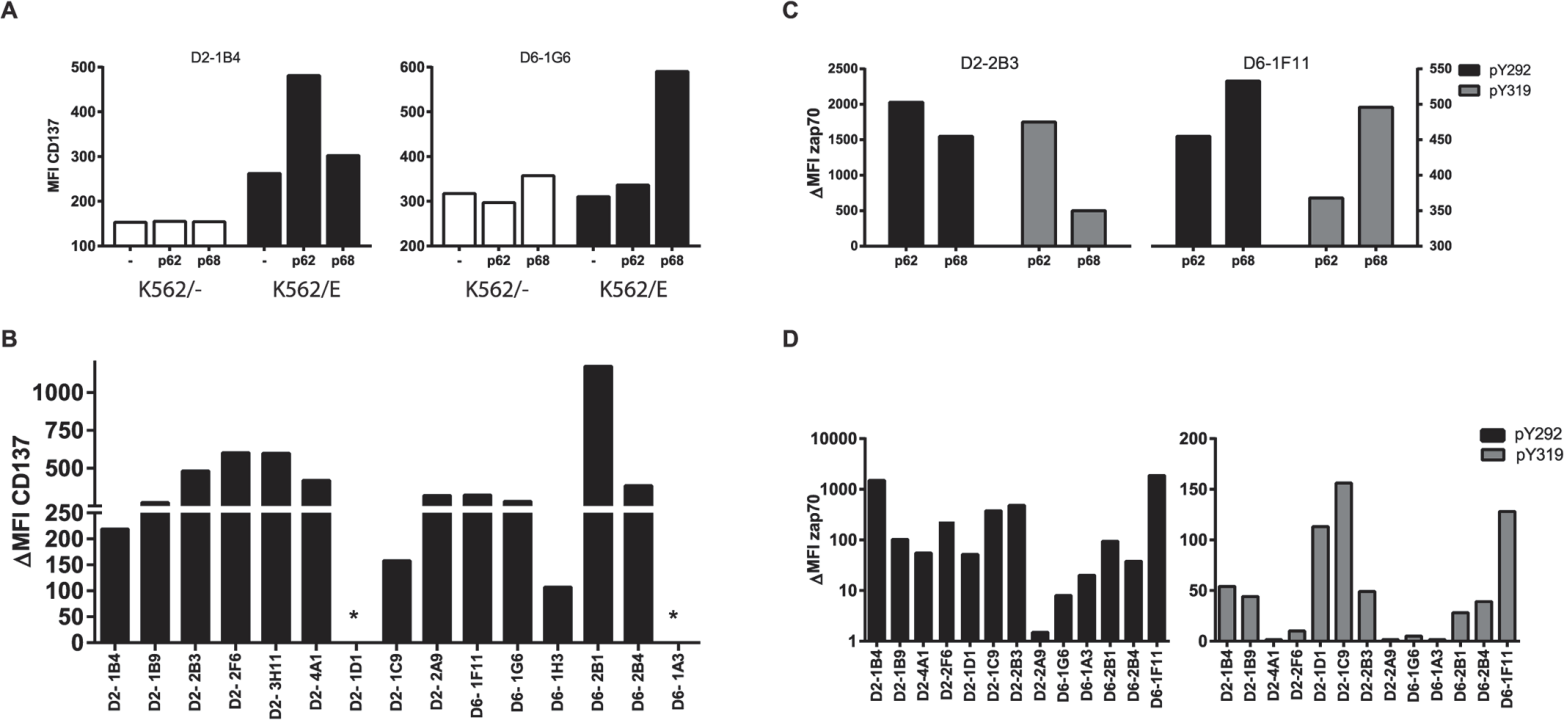
Peptide specific HLA-E restricted CD8^+^ T-cell clones are activated through TCR ligation by specific peptide/HLA-E. A. T-cell clones were stimulated for 16 hours with K562 cells either expressing or lacking HLA-E, which had been pre-loaded with the specific or control peptide. Induction of CD137 expression was determined by flow cytometry. T-cell clones upregulate CD137 only in response to specific but not control Mtb peptide presented in the context of HLA-E. Left panel, T-cell clone specific for peptide 62; Right panel, T-cell clone specific for peptide 68. B. Summary of CD137 upregulation for all T-cell clones tested: T-cell clones from donor 2 recognize peptide 62, whereas T-cell clones from donor 6 recognized peptide 68. Data are expressed as delta MFI, which was calculated by substraction of the MFI obtained with non-peptide pulsed K562/HLA-E from the specific peptide pulsed K562/HLA-E stimulated samples. * indicates no CD137 expression by these T-cell clones despite repetitive measurements. C. T-cell clones were stimulated for 5 minutes with peptide pulsed HLA-E expressing Meljuso cells and stained for 2 different phosphorylation sites of ZAP70 (Y292 in black, Y319 in grey) as indicator of TCR activation. Left panel, T-cell clone specific for peptide 62; Right panel, T-cell clone specific for peptide 68. D. Summary of ZAP70 phosphorylation for all T-cell clones tested for Y292 (left) and Y319 (right). Data are expressed as delta MFI, which was calculated by substraction of the MFI obtained with control-peptide pulsed Meljuso from the specific peptide pulsed Meljuso samples.

Antigen-specific CD8^+^ T-cells induce the expression of CD137 (4-1BB) upon TCR ligation by specific peptide/HLA complexes [26]. Following stimulation with their specific peptide in the presence of HLA-E, upregulation of cell surface CD137 was observed for 14/16 T-cell clones, but not following peptide presentation in the absence of HLA-E or following control peptide presentation, demonstrating specific T-cell activation by antigen presented via HLA-E (Fig. 1A, 1B). A well-known early event following TCR activation is phosphorylation of the TCR associated signalling molecule ZAP70. This occurs within seconds to minutes after TCR mediated recognition of peptide, does not require co-stimulatory signals and is one of the first cellular events following TCR activation. Analysing two independent ZAP70 sites (ZAP70 Y292 and Y319) ZAP70 phosphorylation was found in 11/13 T-cell clones following peptide/HLA-E recognition, thus demonstrating TCR mediated T-cell activation in response to specific peptide presented by HLA-E (Fig. 1C,1D).

### Peptide specific HLA-E restricted CD8^+^ T-cell clones have suppressor or cytolytic effector functions

**Fig 2:**
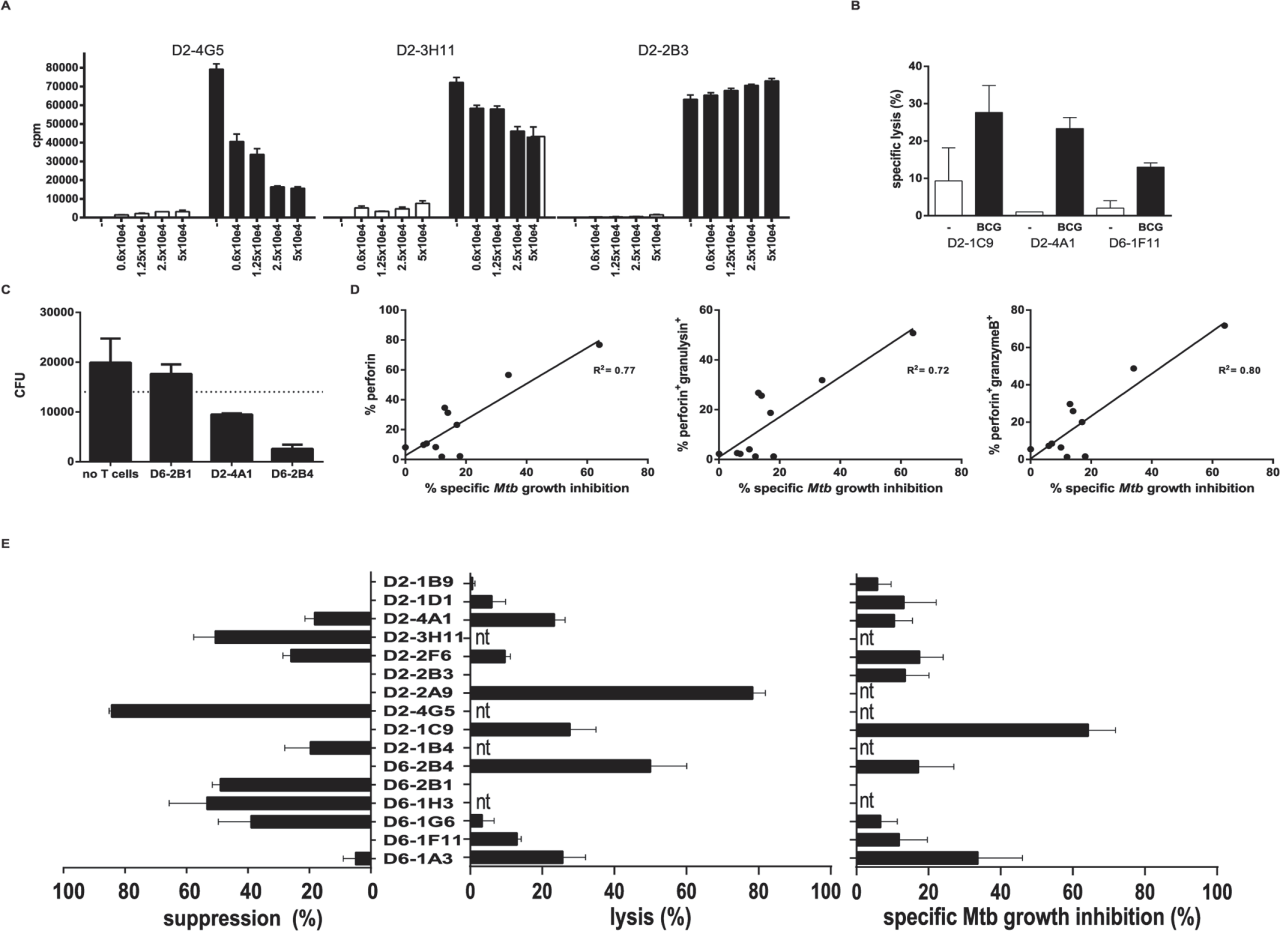
HLA-E restricted T-cell clones possess either suppressive or cytolytic activity. A. T-cell clones were expanded, after which they were added in different ratios to an unrelated reporter Th1 cell clone (Rp15 1-1; Mtb hsp65 p3–13 specific, HLA-DR3 restricted) in the presence of irradiated HLA-DR3 expressing PBMCs as antigen presenting cells together with the cognate peptide recognized by Rp15-1-1 (closed bars). After 3 days of co-culture the proliferative response of the Th1 clone was determined by 3H-TdR incorporation. There was no proliferation in the absence of the cognate p3–13 peptide stimulating the Th1 clone (open bars). Data are expressed in counts per minute (CPM), averaged for triplicate wells (+/- standard deviation). B. T-cell clones were titrated onto ^51^Cr labelled adherent HLA-A2 negative monocytes that were infected with live BCG, and the release of ^51^Cr was determined after 5 hours. Data are expressed as percentage specific lysis. Black bars represent BCG infected monocytes, open bars represent uninfected control monocytes. A ratio of 10:1 (T-cells: monocytes) is shown here. T-cell clones from donor 2 (peptide 62 specific) and from donor 6 (peptide 68 specific) specifically lysed BCG infected target cells. Data represent the average +/- standard deviation of triplicate wells. C. Combined analyses of suppressive and cytolytic activity for all clones tested. The percentage of suppression was calculated by dividing deltaCPM (CPM in presence of p3–13 to activate Rp15 1-1 proliferation minus CPM in absence of p3–13) of 5x10e4 T-cell clones by the deltaCPM of the Th1 clone in the absence of HLA-E restricted T-cell clones (left panel) as described in [58]. Similarly, the percentage specific lysis was plotted in the middle panel. The percentage of specific Mtb killing for each individual clone is plotted in the right panel. The percentage of Mtb killing was calculated after subtraction of the average experimental variation within each experiment and was tested in 3–4 different macrophage donors for each T-cell clone. Nt = not tested. D. T-cell clones were added to Mtb (H37Rv) infected HLA-A2 negative macrophages for 24 hours in a ratio of 5:1 (T-cells: monocytes), subsequently macrophages were lysed and plated for assessment of colony forming units (CFU). CFU were counted and are expressed as CFU/ml lysate. Data represent the average +/- standard deviation of duplicate wells. E. The percentage of intracellular Mtb growth inhibition was calculated by dividing CFU outgrowth from infected macrophages with and without the addition of T-cells for each individual clone. All clones were tested in duplicate in at least 3 independent experiments, using independent macrophage donors. The percentage Mtb growth inhibition was expressed as average of these experiments. The percentage of Mtb growth inhibition was plotted against the percentage of CD8^+^ T-cells expressing perforin (left), perforin and granulysin (middle) and perforin and granzyme B (right), as assessed by flow cytometry. Linear regression analysis was performed to obtain an R^2^ value.

Qa-1 restricted murine CD8^+^ T-cells have been associated with suppressor functions [16,27,28], and we have previously reported a similar phenotype for human HLA-E restricted CD8^+^ T-cells [23]. Classical CD8^+^ T-cells are best known for their cytolytic capacity, and in line with this we previously showed that HLA-E restricted CD8^+^ T-cell lines not only had suppressive but also cytolytic functions [23]. To dissect these rather opposing functions in more detail we tested this at the single cell level in our panel of T-cell clones using highly standardized functional assays. Phenotypic analysis for both regulatory and cytolytic markers revealed mixed patterns. Most clones expressed markers associated with suppressor function, including CD25, LAG3 and CD39, however expression levels of these markers were highly variable (Table 1, S1 Table and S1B Fig.). In addition, several but not all CD8^+^ HLA-E restricted T-cells were able to exert dose-dependent suppression of the proliferative response of an independent reporter Th1 T-cell clone; Fig. 2A shows representative examples of clones with strong (left), intermediate (middle) and no suppressor activity (Fig. 2A). The suppression of proliferation correlated well with the inhibition of IFNγ secretion by the reporter Th1 clone. Although the level of suppression varied amongst the clones that were suppressive, 8/16 clones suppressed Th1 T-cell proliferation clearly and dose-dependently (Fig. 2C left panel, S1 Table and S2 Fig.), in line with our previous results using bulk populations of T-cells.

We next tested whether the HLA-E restricted CD8^+^ T-cell clones had cytolytic activity. Indeed, BCG infected monocytes could be lysed by several but not all clones (Fig. 2B): 7 out of 12 clones tested had this cytolytic activity. Interestingly, 3 out of these 7 clones were able to lyse macrophages in the absence of BCG infection reproducibly (S1 Table, specific lysis indicated between brackets). Moreover, 3 clones did not show any cytolytic nor suppressive activity, whereas 7 clones had either suppressive or cytolytic activity (Fig. 2C and S2 Fig.).

### HLA-E restricted T-cell clones inhibit intracellular Mtb growth

In addition to target cell lysis, and probably of more relevance in the control of intracellular Mtb, we assessed the capacity of our T-cell clones to inhibit outgrowth of intracellular Mtb. To this end we added the T-cell clones to Mtb infected macrophages and lysed them after 24 hours of co-culture after which Mtb was plated to assess the number of CFU. The majority (8/11) of the HLA-E restricted Mtb specific CD8^+^ T-cell clones had the capacity to inhibit Mtb outgrowth, whereas the 3 others did not affect (nor promote) Mtb outgrowth (Fig. 2D). The level of Mtb growth inhibition differed amongst the clones (Fig. 2C, right panel; S1 Table and S2 Fig.), and the magnitude of Mtb growth inhibition was found to correlate with the expression of perforin, perforin and granulysin or perforin and granzyme B (but not granzyme B or granulysin in the absence of perforin, S1 Table) expressed by the T-cell clones (Fig. 2E).

Taken together, the combined results suggest that the majority of the Mtb peptide specific HLA-E restricted human CD8^+^ T-cell clones has either cytolytic and Mtb inhibitory, or alternatively immune-regulatory functionality, whereas a minority of the cells was found to displayed dual functionality.

### Peptide specific HLA-E restricted CD8^+^ T-cell clones have characteristics of Th2 cells

**Fig 3:**
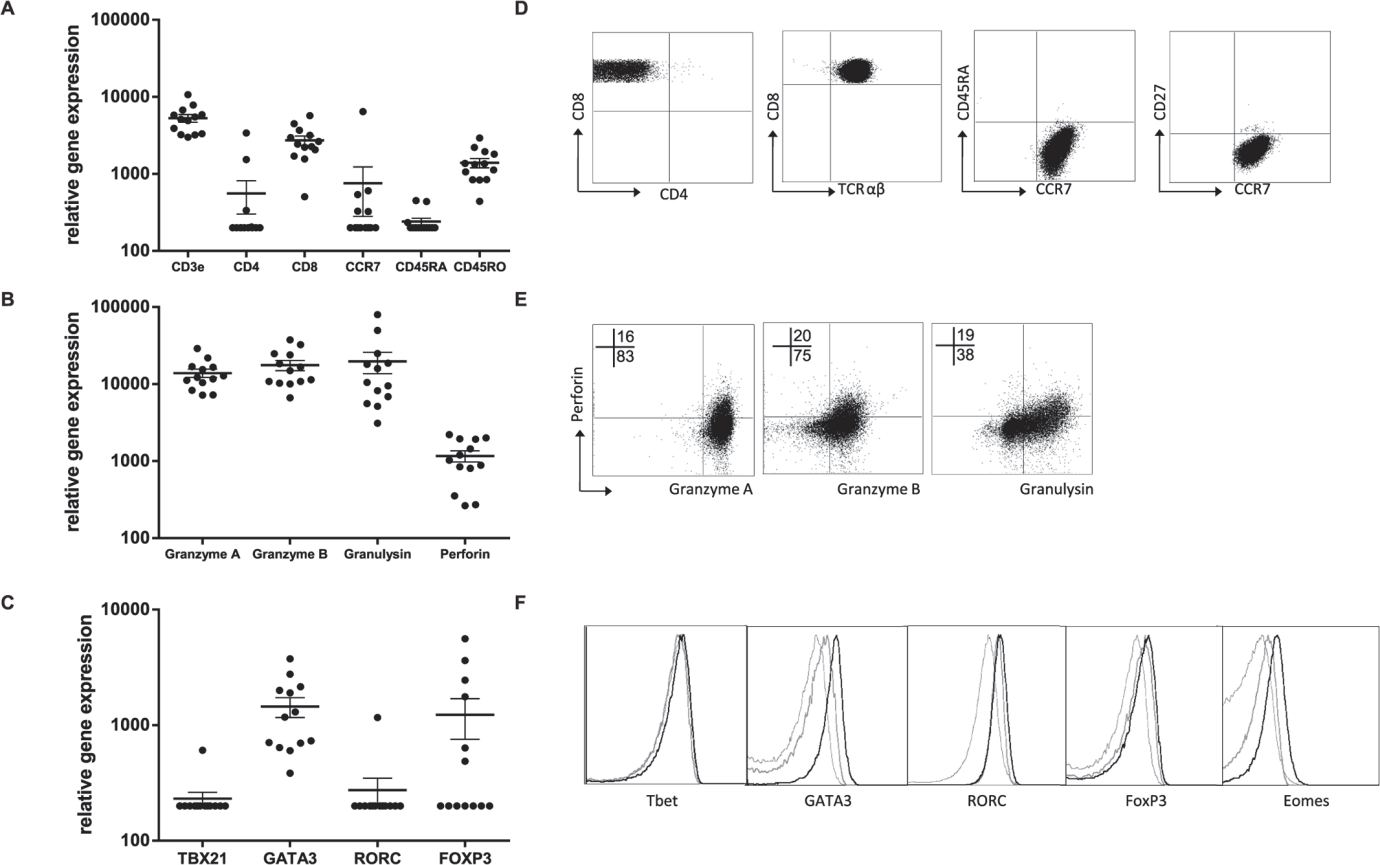
Peptide specific HLA-E restricted CD8^+^ T-cell clones have an effector memory phenotype. T cell clones were cultured in the absence of peptide specific stimulation and RNA was isolated from T-cell clones, RNA expression profiles were determined using dcRT-MLPA and data were normalized for GAPDH expression within each sample (A-C). A. RNA expression levels of classical cellular subset and memory markers of T-cell clones; B. RNA expression levels of cytotoxic effector function associated molecules; C. RNA expression levels of lineage associated transcription factors. D. Flow cytometric analysis of T-cell phenotype, T cell clones were directly stained from culture, a representative T-cell clone is shown (D6-2B4). Gating strategy in S1 Fig. E. Flow cytometric analysis of effector molecules, T-cell clones activated with αCD3/28 beads for 24 hours followed by intracellular staining, a representative T-cell clone is shown (D6-2B4). F. Flow cytometric analysis of lineage determining transcription factors, T-cell clones were directly stained from culture using intracellular staining protocols. Dashed lines represent transcription factor staining in PBMCs, grey (D2–1B9) and black are examples of different T-cell clones (D2-4A1, D6-1F11, D2-2A9).

The T-cell clones were further characterized by assessing RNA expression levels, cell associated (surface) markers and intracellular cytokines as well as secreted cytokines/ chemokines in supernatants. RNA expression analysis using dcRT-MLPA [29] confirmed CD3 and CD8 expression in the absence of CD4 (Fig. 3A). Consistent with the expected phenotype of cloned T-cells they lacked CD45RA, but expressed CD45RO and CCR7, compatible with an effector memory phenotype (Fig. 3D, Table 1). Moreover, as expected for CD8^+^ effector memory T-cells, cytolytic effector molecules granzyme A and B, granulysin and perforin were abundantly expressed (Fig. 3B). The percentage of perforin positive cells was low compared to the percentage of cells expressing other cytolytic molecules, both at the level of RNA as well as by intracellular staining (Fig. 3E), suggesting this to be a possible rate-limiting factor in cytolysis. Interestingly, when lineage determining transcription factors were assessed, we observed no Tbet (TBX21) or RORC, but unexpectedly GATA3 expression was detected in all clones tested (Fig. 3C). Transcription factor expression patterns and lineage determination were confirmed by flow-cytometry (Fig. 3F, Table 1). In addition, intracellular staining revealed eomes expression in 9/13 clones tested, in line with its critical role in the differentiation of effector CD8^+^ T-cells [30] (Fig. 3F, Table 1). Although FOXP3 mRNA was detected in 4/13 clones analysed, intracellular staining did not demonstrate any detectable protein expression (Fig. 3C, Table 1).

**Fig 4:**
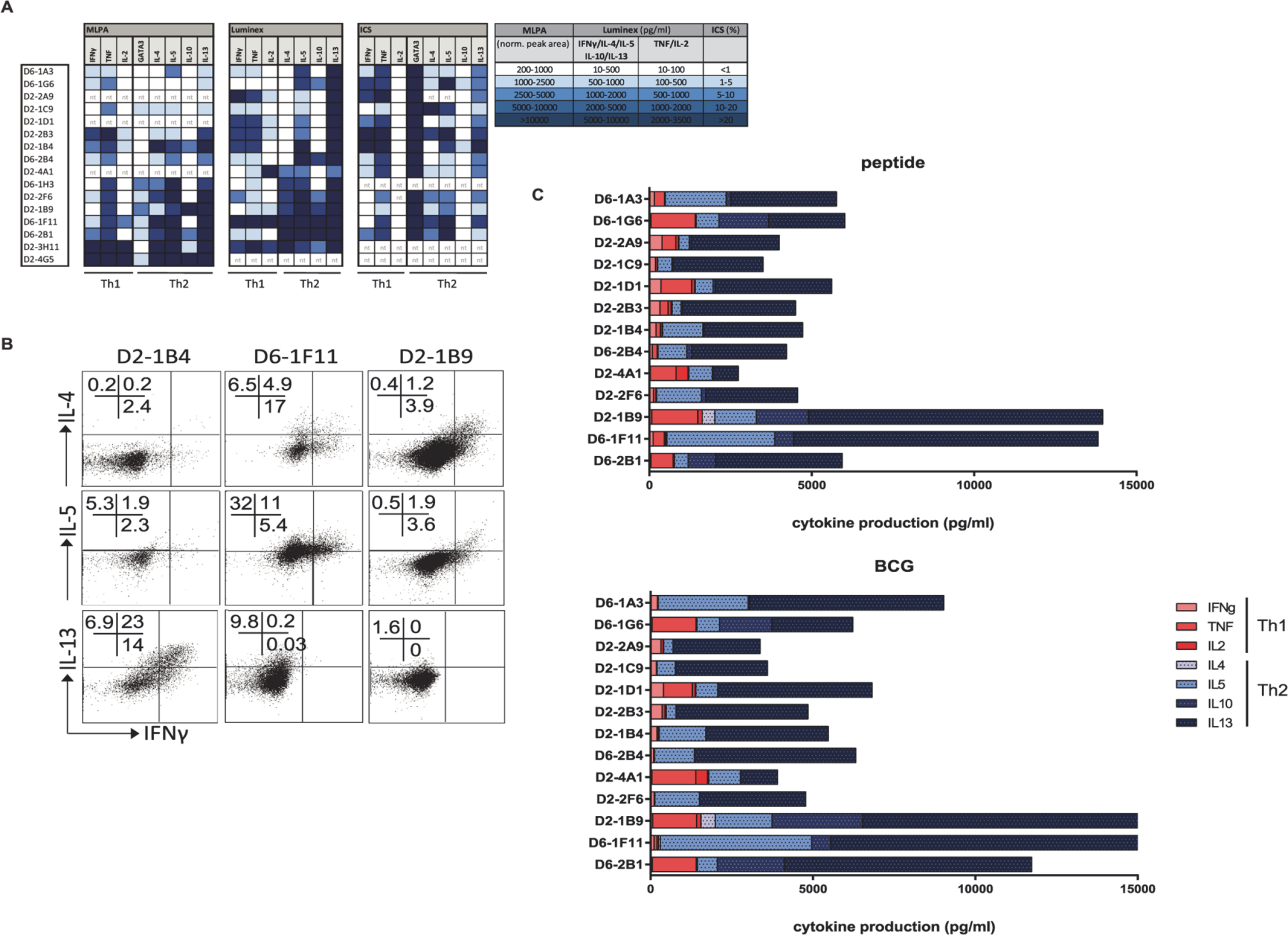
Mtb specific HLA-E restricted T-cell clones produce Th2 cytokines. A. T-cell clones were cultured and RNA was isolated for gene-expression measurement using dcRT-MLPA, data are normalized to GAPDH as housekeeping gene (left panel); clones were stimulated for 24 hours with αCD3/28 beads before supernatants were collected to determine their maximum cytokine secretion profiles (in pg/ml) using multiplex bead arrays (middle panel); similarly, cells were stimulated for 16 hours with αCD3/28 beads in the presence of brefeldin A followed by intracellular cytokine staining to determine intracellular cytokine levels (right panel). Data are expressed as % of the CD3^+^CD8^+^ T-cell population. Data are coloured according to the amount of the molecules detected, according to the legend in the figure. Nt = not tested. B. HLA-E restricted CD8^+^ T-cell clones were stimulated for 24 hours with αCD3/28 T-cell activator beads and stained intracellular for IL-4 or IL-5 or IL-13 as well as IFN-γ. C. T-cell clones were cultured with peptide pulsed macrophages to assess their specific cytokine production in response to peptide presented by professional antigen presenting cells (top panel), or with BCG infected macrophages to assess their specific cytokine production induced by naturally presented antigen during in vitro mycobacterial infection (bottom panel). Supernatants were collected and cytokine/ chemokine levels were determined using multiplex bead arrays.

Because GATA3 expression is associated with Th2 function, we next assessed the production of Th1 and Th2 family cytokines at the RNA and protein level, both using intracellular cytokine staining for flow cytometry and as secreted cytokines in supernatants. The HLA-E restricted CD8^+^ T-cell clones were capable of producing Th1 cytokines, including IFNγ (ranging 70–8754 pg/ml following maximal stimulation using αCD3/28 beads; median 856 pg/ml) and TNFα (ranging 51–2826 pg/ml following maximal stimulation using αCD3/28 beads; median 393 pg/ml) (Fig. 4A), however, we only observed secretion of IFNγ when clones were stimulated maximally using αCD3/28 beads but not when stimulated with specific peptide or with human macrophages infected with BCG (Fig. 4B, C). Thus, although these T-cell clones can be forced to produce Th1 cytokines under maximal, non-physiological stimulation and co-stimulation conditions, they fail to do so when stimulated under physiological conditions when seeing antigen (including macrophages infected with mycobacteria) (Fig. 4C).

All CD8^+^ T-cell clones evaluated were able to produce Th2 cytokines, as measured in supernatants following maximal stimulation with αCD3/28. All clones produced IL-13 and IL-5 whereas 6/15 clones also produced IL-4 (Fig. 4A, middle part). Secreted levels of IL-13 following maximum stimulation ranged from 1763–10.000 pg/ml, with a median production of 9153 pg/ml. IL-13 was already secreted at high steady state levels by the T-cell clones in the absence of additional stimulation, but in the presence of macrophages (median 3471 pg/ml) and these levels did not increase considerably by stimulation with either peptide loaded or BCG infected macrophages (Fig. 4C, S1 Table). IL-4 and IL-10 levels varied among the clones: 6/15 clones produced IL-4 and 7/15 produced IL-10 (mostly overlapping patterns) whereas another 6 clones produced neither IL-4 nor IL-10 (Fig. 4A). Interestingly, clones that secreted most IFN-γ and TNF (following stimulation with αCD3/28 beads) did not secrete IL-4 and IL-10, suggesting some sub-dichotomy in Th1/ Th2 patterns for these CD8^+^ T-cell clones (Fig. 4A). Intracellular cytokine staining confirmed IL-4, IL-5 and IL-13 cytokine production by the CD8^+^ T-cell clones (Fig. 4B, S1 Table).

Cytokine production in response to specific peptide stimulation indicated an even stronger Th2 profile, with a virtual absence of any Th1 cytokine production (Fig. 4C). Moreover, the same cytokine production patterns were also seen when cells were stimulated with BCG infected macrophages (Fig. 4C, right panel and S2 Fig.) indicating that natural processing and presentation of Mtb epitopes via HLA-E activates CD8^+^ T-cell type 2 cytokine production.

### HLA-E restricted CD8^+^ T-cells can activate B-cells by production of IL-4

**Fig 5:**
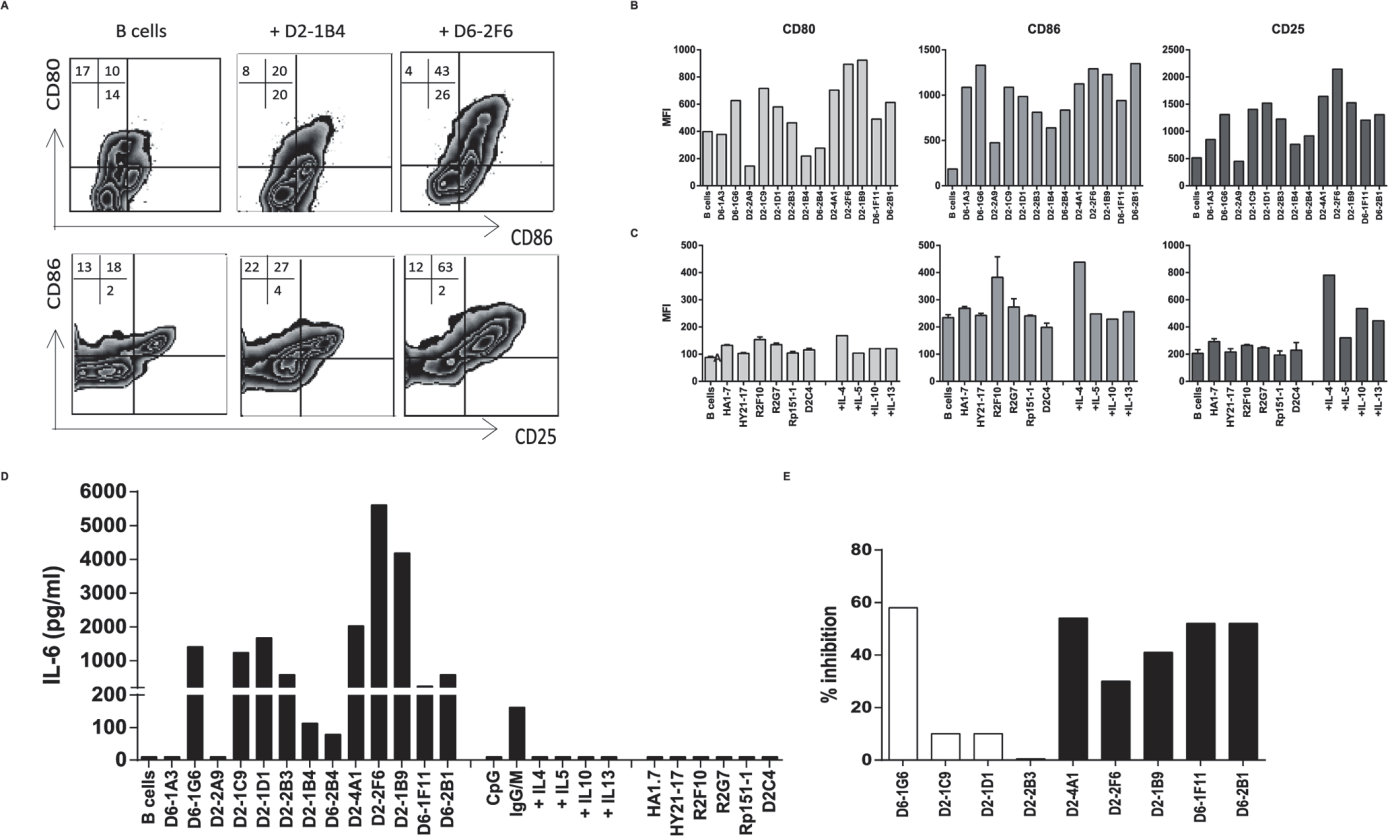
HLA-E restricted Mtb specific T-cell clones utilize IL-4 to provide B-cell help. T cell clones were co-cultured with primary CD19^+^ B-cells in a 1:1 ratio for 48 hours, subsequently B-cell activation was determined by flow cytometry and by measurement of IL-6 in supernatants. A. Flow cytometric analysis of B-cells only (top row), or B-cells co-cultured with 2 independent T-cell clones and stained for CD80, CD86, and CD25. Cells are gated on CD3^−^CD19^+^ cells. B. B-cell activation induced by HLA-E restricted Mtb specific T-cell clones as indicated by expression of CD80, CD86, and CD25. C. B-cell activation induced by panel of unrelated, (CD4^+^) control T-cell clones and by recombinant cytokines. B-cell activation is assessed by flow cytometry. D. IL-6 production in supernatants of co-cultures of B-cells with HLA-E restricted Mtb specific CD8^+^ T-cells, B-cell activators (CpG, αIgG/M), recombinant cytokines and unrelated control T-cell clones. Data are expressed as pg/ml in supernatant. E. Co-culture of B-cells with HLA-E restricted Mtb specific T-cell clones in the presence of blocking antibodies against IL-4, IL-5 or IL-13, supernatants were collected and IL-6 measured by ELISA. Data are expressed as percentage inhibition of IL-6 production in supernatants of specific antibody blocking compared to the isotype control.

Since the HLA-E restricted Mtb specific T-cell clones produced IL-5 and IL-13, and some clones also produced additional IL-4 and IL-10, we also investigated B-cell activation. Indeed, HLA-E restricted T-cell clones co-cultured with CD19^+^ B-cells induced increased expression of CD25, CD80, CD86 (Fig. 5A) and HLA-DR (S1 Table) on B-cells, indicating B-cell activation. Most HLA-E restricted Mtb specific CD8^+^ T-cell clones were in fact able to induce increased expression of CD80, CD86 and CD25 on CD19^+^ B-cells (Fig. 5B). Classical CD4^+^ and CD8^+^ T-cell clones did not activate B-cells (Fig. 5C), suggesting that a specific property of HLA-E restricted CD8^+^ T-cells is the ability to activate for B-cells. Only the classical HLA class II restricted CD4^+^ T-cell clone R2F10 induced some B-cell activation, but this clone is known to produce IL-4 [31]. Since the HLA-E restricted CD8^+^ T-cell clones expressed various Th2 cytokines, we verified, using recombinant cytokines, that recombinant IL-4 induced the strongest upregulation of CD80, CD86 and CD25 (Fig. 5C).

Activated B-cells produce and secrete IL-6 thus IL-6 in supernatants is considered an important hallmark of B-cell activation. Our T-cell clones did not produce IL-6, even when stimulated maximally with αCD3/28 beads (S1 Table). Supernatants collected from co-cultures of 11/13 HLA-E restricted CD8^+^ T-cells with primary B-cells contained IL-6 (Fig. 5D), demonstrating B-cell activation. In contrast, supernatants from B-cells co-cultured with control T-cell clones did not contain IL-6 (Fig. 5D). To further confirm the key cytokines involved in B-cell activation we added blocking antibodies to the co-cultures of HLA-E restricted Mtb specific T-cell clones that were capable of activating B-cells (IL-6 > 200 pg/ml & IL-4 production by T-cell clone > 1000 pg/ml) with B-cells and measured IL-6 levels in supernatants. Blocking antibodies to IL-4 inhibited IL-6 in supernatants of co-cultures, whereas antibodies to IL-5 or IL-13 did not inhibit B-cell secreted IL-6, indicating that T-cell derived IL-4 is responsible for B-cell activation (Fig. 5E).

### HLA-E restricted Mtb specific T-cells are present in TB patients and produce Th2 cytokines upon peptide stimulation

**Fig 6:**
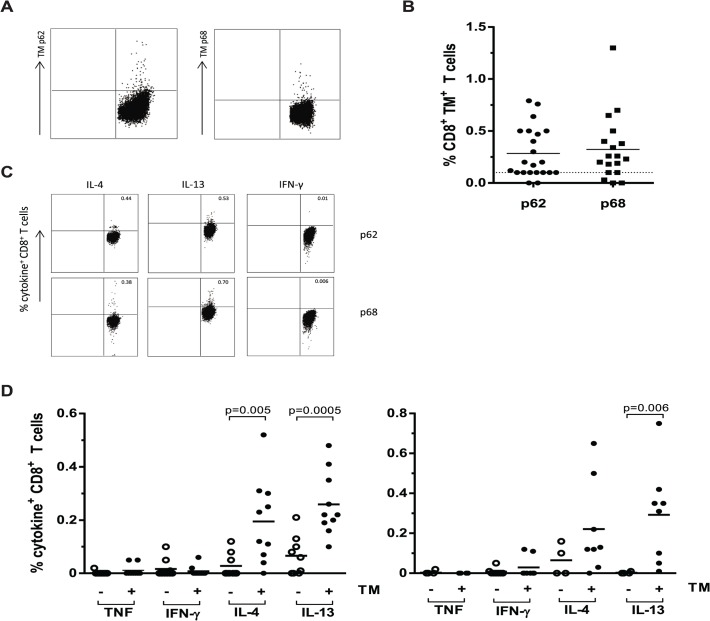
CD8^+^ T-cells from TB patients bind HLA-E/ peptide tetramers and produce Th2 cytokines following peptide stimulation. PBMCs from patients with pulmonary TB were stained directly *ex vivo* with HLA-E/ peptide tetramers and analysed by flow cytometry. Data are expressed as the percentage tetramer positive cells within the CD8^+^ population. PBMCs were stimulation with either peptide 62 or peptide 68 for 16 hours in the presence of monensin. Cytokines were stained by intracellular staining followed by flow cytometric analysis, data are expressed as percentage of CD8^+^ T-cells. A. Example flow cytometry results for a representative single TB patient following staining with HLA-E tetramers containing peptide 62 or peptide 68, cells are gated on CD8^+^ T-cells. B. Results of combined TM staining on PBMCs from TB patients for both tetramers containing P62 or P68, data are expressed as percentage of CD8^+^ T-cells. C. Example of intracellular cytokine staining following peptide stimulation for a single representative TB patient, cells are gated on CD8^+^ T-cells. D. Cytokine production by CD8^+^ T-cells following stimulation with peptide 62 (left) and peptide 68 (right). Open circles represent patients with tetramer staining <0.1%, close circles represent patients with tetramer staining >0.1%. Groups were compared using a Mann-Whitney U test and p<0.05 was considered significant.

Finally, it was important to assess whether these HLA-E restricted *Mtb* specific T-cells were also present in the circulation of TB patients during active infection. T-cells recognizing these peptides were detectable in the blood of active TB patients directly *ex vivo* using HLA-E/peptide tetramers (Fig. 6A). HLA-E tetramers containing peptide 62 were recognized by 13 out of 22 TB patients (having more than 0.1% tetramer positive CD8^+^ T-cells) with an average of 0.28% (range 0.12–0.76%)(Fig. 6B). Moreover, HLA-E tetramers containing peptide 68 were recognized by 13 out of 18 TB patients, with an average of 0.32% of CD8^+^ T-cells (range 0.18–1.3%)(Fig. 6B). Tetramer responses were absent in healthy uninfected (PPD negative) individuals, in accordance with our previous data [23].

We then studied the capacity of these peptides to stimulate cytokine production following overnight peptide stimulation of PBMCs of all TB patients (Fig. 6C). In agreement with the above results from the T-cell clones, peptide stimulated PBMC from TB patients showed little or no TNF-α and IFN-γ production but produced significant levels of IL-4 and IL-13, especially in the tetramer positive group (Fig. 6D; using an arbitrary cut-off of tetramer responses > 0.10% as positive). Peptide 62 induced IL-4 and IL-13 responses by CD8^+^ T-cells that were significantly higher in the tetramer positive donors compared to the tetramer negative donors. Peptide 68 induced significantly increased IL-13 secretion by CD8^+^ T-cells of tetramer positive TB patients. For both peptides 62 and 68 all tetramer positive TB patients were capable of producing either IL-4 or IL-13 and for peptide 62, 7 out of 10 patients produced both cytokines with levels higher than 0.1% cytokine producing T-cells. For peptide 68 the number of patients that was able to produce both cytokines upon the peptide stimulation was even as high as 6 out of 8 (Fig. 6D).

Taken together these findings demonstrate the presence of HLA-E restricted *Mtb* peptide specific T-cells in the circulation of TB patients. Moreover, they confirm the strong Th2 profile of the response against these peptides in active infection, in concordance with the phenotype of the HLA-E restricted, peptide specific CD8^+^ T-cell clones.

## Discussion

We here report that non classical, Mtb peptide reactive HLA-E restricted CD8^+^ human T-cells have an unorthodox phenotype: in contrast to classical Mtb induced CD4^+^ and CD8^+^ T-cells that are mostly Th1 type cells, these HLA-E restricted T-cells expressed GATA3, predominantly produced the Th2 cytokines IL-4,-5,-10 and -13, were able to exert either cytolytic or suppressive functions, and also provided B-cell help through IL-4. Contrary to the dogma that Th2 cells may negatively impact on control of Mtb, these non-classical HLA-E restricted T-cell clones were able to inhibit intracellular Mtb growth, which correlated to their expression of the cytolytic molecules granzyme, perforin and granulysin. Moreover, patients with pulmonary TB had such ‘Th2-like’ non classical CD8^+^ T-cells in their circulation as visualised using specific Mtb peptide/HLA-E tetramers. Thus our results identify a new, non-classical, multifunctional T-cell subset which is engaged in the human immune response to infection, including during active TB. These results significantly expand our understanding of the human immune response to infection.

Interestingly, all HLA-E restricted CD8^+^ T-cell clones we analysed expressed the Th2-associated transcription factor GATA3 and produced the Th2 cytokines IL-5 and IL-13. Moreover, IL-4 and IL-10 were produced by half of the clones. GATA3, classically known as transcription factor for Th2 differentiation, functions as a chromatin-remodelling factor for the IL-4, IL-5 and IL-13 locus and can act as transcription factor for IL-5 and IL-13 genes [32]. Besides transcriptionally regulating expression of Th2 related cytokines, GATA3 also appears crucial for peripheral maintenance and proliferation of murine CD8^+^ T-cells [33,34]. Indeed, in murine LCMV infection and during alloantigen triggered graft versus host responses, GATA3 deficiency resulted in poor antigen specific T-cell proliferation [33]. In addition, GATA3 deficient mice have an impaired ability to kill tumour cells by CD8^+^ T-cells, indicating that GATA3 expression is required to mediate fully efficient cytolytic effector responses [34]. This agrees with our own observations here in that all of our cytolytic CD8^+^ T-cell clones expressed GATA3. More importantly, the results document the involvement of GATA3 in anti-mycobacterial immunity for the first time, highlighting Th2 cytokine production, B-cell help and cytolysis as relevant GATA3 related functions in the immune response to mycobacteria.

GATA3 expression levels within CD8^+^ T-cells seem a proper biomarker of immune dysfunction in patients with systemic sclerosis, a connective tissue disorder involving multiple organs [35]. As expected, the percentage of GATA3^+^ CD8^+^ T-cells correlated with the percentage of IL-13^+^ CD8^+^ T-cells, which are thought to play an important role in tissue fibrosis. Indeed in several skin disorders (atopic dermatitis, psoriasis, sclerosis) associated with fibrotic responses and inflammation, locally increased CD8^+^ IL-13 producing T-cells have been identified that seemed critical players [36–38]. Interestingly, the percentage of GATA3^+^ CD8^+^ T-cells was highest in the systemic sclerosis patients that also had interstitial lung disease, characterised by inflammation and fibrosis [35]. Moreover, also in human allergic asthma, IL-13 producing CD8^+^ T-cells isolated from the lung are increased and associated with airway obstruction [39]. Together these studies suggest that IL-13 producing CD8^+^ T-cells are important players in inflammatory disorders in the lung.

Recently Th2 responses have been associated with TB in a number of studies. In zebrafish, mycobacterial infection induced Th2 gene expression signatures were associated with lower bacterial burdens, suggesting a contribution of Th2 immunity to control of mycobacterial infections [40]. In mice, Th2 responses during TB disease were initially considered to abrogate protection through inhibition of Th1 immunity, however, infection experiments in mice incapable of mounting Th2 responses have demonstrated that Th2 responses are not responsible for the inability to control Mtb infection [41]. However, Th2 immunity, such as IL-4 and IL-13 may be involved in disease related pathology, since overexpression of IL-13 in a murine Mtb infection model resulted in enhanced pathology, mimicking the human TB lesions closely [42]. In humans, mRNA levels for IL-13 were increased in Mtb infected lymph nodes, indicating local IL-13 expression in Mtb lesions, although the cellular source remained undefined [43]. BAL and plasma of patients with pulmonary TB contained increased levels of IL-4 compared to patients with other lung diseases, patients with moderate-advanced TB had higher levels of IL-4 compared to patients with mild TB disease [44]. Moreover, IL-4 producing T-cells have been found in the circulation of patients with pulmonary TB at diagnosis that disappeared rapidly following initiation of chemotherapy [45]. Here we now also show that patients with active TB have CD8^+^ T-cells that recognize Mtb derived peptides in the context of HLA-E, based on peptide/HLA-E tetramer staining. These results were further strengthened by peptide stimulation experiments that again confirmed the presence of a Th2 type response to these Mtb ligands.

In contrast to previous hypotheses that postulated that Th1 but not Th2 were involved in controlling intracellular pathogens like Mtb, we show here that also Th2 cytokine producing CD8^+^ T-cells can actively lyse Mtb infected cells and, more importantly, limit intracellular Mtb growth. This is in line with a recent report from zebrafish in which the expression of Th2 cytokines was associated with lower bacterial burdens [40]. In patients, IL-4 producing T-cells were present at diagnosis and correlated with disease severity [44,45], indicating that they are involved in the disease process, but unfortunately the anti-mycobacterial capacity of these cells was not reported. The anti-mycobacterial activity of our HLA-E restricted ‘Th2-like’ CD8^+^ T-cells suggests that these cells may contribute significantly to Mtb inhibition, and constitute a relevant part of the total immune effector repertoire against mycobacteria.

In addition to the direct anti-mycobacterial activity of these ‘Th2-like’ CD8^+^ T-cells, we find that HLA-E restricted Mtb specific CD8^+^ T-cell clones can activate B-cells, with subsequent antibody production as additional component in the combat against Mtb. Recently, there has been a renewed interest in B-cells in TB mostly since B-cell related genes are specifically expressed during TB disease and change during successful treatment [29,46–48]. Experimental data have provided more direct evidence for the importance of B-cells in TB. Firstly, B-cell deficient mice appear more susceptible to TB [49], secondly, B-cell follicle structures and activated B-cells have been found in granulomas of human and nonhuman primates infected with Mtb [50,51]. Thirdly, receptors for B-cell secreted immunoglobulins, and in particular the expression of the human Fcγ R1is a consistent and strong component of TB biomarker signatures [29,52–54]. In addition, recently a cytosolic Fc-receptor called TRIM21 [55,56], was identified that can bind intracellular complexes of immunoglobulin bound to pathogen, resulting in subsequent immune activation and inflammation. These observations suggest that B-cells and/or Mtb specific immunoglobulins may play a hitherto unappreciated role in effector responses towards Mtb. The activation of B-cells by the Mtb specific HLA-E restricted CD8^+^ T-cells could be of potential benefit when utilizing this non-classical antigen presentation pathway in TB vaccination strategies, next to its limited allelic variation and its resistance to HIV-nef mediated downregulation.

Mtb-specific HLA-E restricted CD8^+^ T-cells that produce IL-4 and IL-13 may home to the lung and participate in local immune responses during active TB. This could potentially lead to a variety of outcomes, including inhibition of Mtb by cytolysis and inhibition of intracellular Mtb growth, regulation of inflammation, tissue damage and fibrosis. However, Mtb specific HLA-E restricted CD8^+^ T-cells also include T-cells with potent immune-suppressing capacities, suppressing bystander (CD4^+^) T-cell proliferation as well as effector cytokine production. These regulatory functions may dampen local inflammatory responses, particularly since these cells should have co-evolved within the former population of HLA-E restricted T-cells. The balanced induction of both functionally diverse populations may promote balanced inflammation [46]. Balanced immunity is thought to be critical to effectively control infection (protective immunity) as well as hyper- or hypo-inflammation (pathogenic immunity), and needs to be tightly regulated by immune cells participating in host defence to the invading pathogen. Our finding that T-cells recognizing the same Mtb epitopes can have opposing, complementary functionalities in this respect may be relevant in achieving such a balance. It will be critical to further decipher how the balance between these complementary responses is determined before considering application of such peptides as vaccines.

Thus, our data show that non classical human CD8^+^ T-cells recognize Mtb peptides in the context of HLA-E and have strong ‘Th2-like’ characteristics, can activate B-cells through IL-4, and can either lyse infected target cells, inhibit intracellular Mtb growth or regulate inflammatory Th1 responses. These results reveal a novel, non-classical T-cell subset in humans which is engaged in the immune response to infection.

## Materials and Methods

### Ethics statement

T-cell lines were generated from healthy anonymous bloodbank donors (Sanquin Bloodbank, the Netherlands) that had signed written informed consent for scientific use of blood products, donors 2 and 6 were the same donors as previously published [23].

Collection of PBMCs from TB patients collected at the University of Palermo, Italy was approved by the Ethical Committee of the University Hospital, Palermo, where the patients were recruited. The study was performed in accordance to the principles of the Helsinki declaration and those of the “Good Clinical Practices”, and all individuals gave written informed consent to participate.

### Donor and peptide information

Peptide #62 (RMPPLGHEL, Rv2997, accession number O53244) and #68 (VLRPGGHFL, Rv1523,accession number Q50584)[23] were purchased from peptide2.0 Inc (Chantilly, VA, USA). CD8^+^ T-cells from donor 2 recognized peptide #62, but not #68, whereas T-cells from donor 6 recognized peptide #68 but not #62. In all experiments involving peptide recognition, specific and control peptide were compared. Control peptide in each case was the alternative peptide recognized by the other donor.

Materials and methods are written in consensus with the most recent MIATA guideline (minimal information about T-cell assays) when applicable [25].

### Generation of HLA-E restricted T-cell clones

T-cell lines were generated by stimulation of PBMCs with peptide (10μg/ml) for 2 weeks, followed by purification of CD8^+^ cells using magnetic beads (Milteny Biotec BV, Leiden, The Netherlands). Lines (2x10e5 c/w) were cultured in Iscove’s modified Dulbecco’s medium (IMDM, Gibco Life technologies, Thermo Fisher Scientific Inc, Merelbeke, Belgium), supplemented with 10% pooled human serum and were restimulated in 96 well round bottom plates with irradiated (30 Gy) pooled (5 donors) PBMCs pre-pulsed with peptide (25 μg/ml, 5x10e5c/w), in the presence of IL-7, IL-15 (both 5 ng/ml, Peprotech, Rocky Hill, NJ) and IL-2 (50U/ml, Proleukin, Chiron, Amsterdam, the Netherlands). Every other day cells were split and fresh IL-2 (100 U/ml) was added.

Cultures of purified CD8^+^ T-cells were incubated during 16 hours with fresh peptide pulsed feeder-cells, subsequently, cells were labelled with CD137-PE (BD Biosciences, Erembodegem, Belgium), followed by incubation with PE beads (Miltenyi Biotec BV, Leiden, the Netherlands) and CD137^+^ cells were isolated. CD137^+^ cells were diluted to 0.3 cells per well and plated in 96 well round bottom plates containing 5x10e5 peptide-pulsed irradiated feeder cells in the presence of IL-2 (50U/ml). After two weeks of culture, growing clones were selected from the 0.3 c/well cultures and expanded as described above with alternating peptide pulsed irradiated feeders or T-cell expander beads. In total 30 clones were isolated, of which 16 randomly selected clones were analysed in detail.

### Flow cytometric analysis

T-cell clones were stained for surface expression, intracellular markers or cytokines; live/dead stain (Vivid fixable violet reactive dye, Invitrogen, Thermo Fisher Scientific Inc, Merelbeke, Belgium) was used for all samples according to the manufacturer’s protocol. T-cell clones were further characterized in detail by cell surface staining directly from culture for CD3-PE-TexasRed (Invitrogen), CD4-PE-Cy5, CD8-HorizonV500, CD94-PerCP-Cy5.5, TCR-αβ-FITC or TCR-γδ-FITC, CD56 PE-Cy7, CD16-BrilliantViolet605, CD127-BrilliantViolet 650 (all BD Biosciences), NKG2A-APC, NKG2C-PE, NKG2D-AlexaFluor700 (R&D Systems, Abingdon, UK), or intracellular with fixation and permeabilization reagents (ADG, ITK Diagnostics, Uithoorn, The Netherlands) for CD3-Alexa700, CD4-PE-Cy7, CD8-HorizonV500, CD27-PE, CD45RA-FITC (all BD Biosciences), CCR7-APC-Cy7, Tbet-BrilliantViolet605 (Biolegend, ITK Diagnostics, Uithoorn, The Netherlands), RORC-APC, GATA3-PerCP-eFluor710, FoxP3-PECy5 and Eomes-PE-C594 (eBioscience, Vienna, Austria). Cytolytic molecules were assessed after a 24 hour stimulation with T-cell expander αCD3/28 beads (Invitrogen) by intracellular staining with rabbit-anti-human Granulysin (kind gift of Dr. A. Krensky, Stanford, CA) followed by Goat-anti-Rabbit-FITC, CD3-PE-Cy5, CD4-PE-Cy7, CD8-HorizonV500, CTLA4-PE-C594, CD25-APC-H7, Perforin-PE, GranzymeB-AlexaFluor700 (all BD Biosciences) LAG3-Atto647 (Enzo Life Sciences BVBA, Raamsdonksveer, the Netherlands), GranzymeA-PerCPCy5.5 (Biolegend). Finally, cytokine profiles of the T-cell clones were analysed after addition of T-cell expander beads for 6 hours followed by 16 hours incubation with BrefeldinA (3 μg/ml, Sigma-Aldrich Chemie BV, Zwijndrecht, the Netherlands). Cells were stained for surface expression of CD3-PE-TexasRed (Invitrogen) CD4-PE-Cy5, CD8-HorizonV500, and intracellular for GATA3-PerCP-eFluor710, TNF-PE-Cy7, IL-2-BrilliantViolet605, IFN-γ-AlexaFluor700, IL-13-PE, IL-4-PE (all BD Biosciences), IL-5-PE (Biolegend), IL-10-APC (Miltenyi) and CCL4-FITC (R&D Systems).

### Analysis of HLA-E restriction and peptide specificity of the T-cell clones

T-cell clones (1x10e5 c/w) were incubated with peptide loaded K562 cells (5x10e4 c/w) with or without the HLA-E allele (kind gift of Dr. E. Weiss, Ludwig-Maximilians-Universität, Munich, Germany) [57] in multiple wells in a 96-well round-bottom plate. Stable surface expression of HLA-E is induced by 24 hour incubation at 26°C followed by peptide loading (20 μg/ml) for 16 hours at 26°C and stabilization at 37°C for at least 2 hours prior to use. After 4–6 hours of co-culture, BrefeldinA (3 μg/ml) was added and cells were incubated for an additional 16 hours before flow cytometric analysis was performed.

Cells were harvested and stained for CD3-PE-TexasRed (Invitrogen), CD4-PE-Cy7, CD8-HorizonV500 (BD Biosciences), followed by intracellular staining for CD137-PE-Cy5 (BD Biosciences) using fix/perm reagents (ADG).

Specific TCR triggering was assessed by phosphorylation of zap70 with Phosflow analysis. T-cell clones (0.5–1x10e6 cells) were incubated in a 24-well plates for 5 minutes at 37°C in the presence of peptide pulsed MelJuSo cells (35000 cells/well, cell line was kindly provided by Prof. J. Neefjes, Dutch Cancer Institute, Amsterdam, the Netherlands). T-cell expander beads (Invitrogen), were used to assess maximum zap70 phosphorylation. After incubation, T-cells were fixed (BD lyse/fix for Phosflow buffer, BD) for 10 minutes at 37°C, permeabilized for 30 minutes at 4°C with perm buffer III and stained for 1 hour at 4°C with the Phosflow reagents according to manufacturer’s protocol (CD3-PE-TexasRed (Invitrogen) CD4-PE-Cy5, CD8-HorizonV500, Zap70-pY292 AlexaFluor647, Zap70-pY319/Syk-pY352 –PE, all BD Biosciences).

Cells were acquired on a LSRFortessa with Diva software (v6.2, BD Biosciences). Analysis was performed with Flowjo software (v9.5.3, Tree Star Inc, Ashland, OR).

### BCG and H37Rv preparation

BCG (Pasteur strain) or Mtb (H37Rv) was grown in Middlebrook 7H9 medium supplemented with 10% ADC (BD Biosciences), log phase bacteria were used for infection experiments. Multiplicity of infection was calculated based on determination of the number of viable bacilli per ml by plating serial dilutions of bacteria on Middlebrook 7H10 agar plates supplemented with 10% OADC (BD Biosciences) and counting of visible colonies after 3 weeks. Infections of monocytes, macrophages and adherent Meljuso cells were done at a MOI of 10.

### Suppression assay

HLA-E restricted peptide specific T-cell clones were tested for their ability to inhibit proliferation of a Th1 responder clone (Rp15 1-1) as previously described [23,58–60]. Rp15 1-1 T-cells (1x10e4 c/w) were cultured in a 96-well flat-bottom plate with irradiated (20 Gy), HLA-DR3 matched PBMCs as antigen presenting cells (5x10e4 c/w) and 0.05–0.1 μg/ml of hsp65 peptide 3–13, specific for the Th1 responder clone, in the absence or presence of HLA-E restricted T-cell clones (0.6–5x10e4 c/w). Proliferation was measured by [3H] TdR incorporation (0.5 μCi/well, Perkin Elmer, Groningen, the Netherlands) after 96 hours. Cells were harvested with a 96-well Tomtec cell harvester (Synchron, Etten-Leur, the Netherlands) and counts per minute (cpm) were determined using a Wallac MicroBeta counter (Perkin Elmer, Groningen, The Netherlands).

### Cytotoxicity analyses

Cytotoxic capacity of the T-cell clones was tested in a standard ^51^Cr release assay [23,58,61]. PBMCs from a HLA-A2 negative (given the possible overlap in peptide binding profiles between HLA-E and HLA-A2 molecules [62]) buffy coat were plated at 1.5x10e5 cell/well in a 96 well flat bottom plate for 5 days. Non adherent cells were washed away and cells were incubated with specific or control peptide (10μg/ml) or were infected with live BCG (MOI of 10) for 8 hours followed by incubation with 1 μCi ^51^Cr for 16 hours. The next day, cells were washed three times and T-cell clones were titrated on the target cells and incubated for 5 hours, followed by measurement of ^51^Cr release on a Wallac Wizard^2^ gamma counter (PerkinElmer). Percentage specific lysis was calculated per well ((sample release/maximum release) *100%).

### Intracellular Mtb growth inhibition assays

Macrophages were generated from CD14^+^ monocytes that were isolated from HLA-A2 negative buffycoat PBMCs with CD14 MACS beads (Miltenyi) and differentiated for 6 days in the presence of 50 ng/ml M-CSF (R&D systems). Macrophages were harvested and seeded at 3x10e5 c/well in a 24 well plate for adherence. After 18 hours macrophages were infected with Mtb H37Rv from a log phase culture at a MOI of 10 for 1 hour followed by three washing steps with culture medium in the presence of gentamycin (Lonza Benelux BV, Breda, the Netherlands) (2 times with 30 μg/ml and once with 5 μg/ml). Infected cells were rested overnight, the next day T-cell clones were added at an E:T ratio of 5:1 in duplicate in the presence of specific peptide. After 24 hours of co-culture, the cells were lysed and serial dilutions were plated on 7H10 agar plates, supplemented with BBL Middlebrook OADC enrichment (BD Biosciences) for Mtb CFU determination. Colonies were counted after two to three weeks incubation at 37°C. Specific intracellular Mtb growth inhibition was calculated per experiment after substraction of the average variation in Mtb CFU in the absence of T-cells, all clones were tested in duplicate in 3–4 independent experiments using unrelated Mf donors and results of these experiments were averaged to obtain the percentage specific Mtb growth inhibition.

### RNA expression profiling by dcRT-MLPA assay

HLA-E restricted T-cell clones (1x10e6 cells) were lysed in TriZol reagent (Invitrogen) and RNA was isolated according to the instructions of the manufacturer. RNA was quantified using a Nanodrop ND-1000 spectrophotometer and diluted to 50 ng/μl for use in dcRT-MLPA.

A dual-colour reverse transcriptase multiplex ligation-dependent probe amplification (dcRT-MLPA) assay was performed as described previously [29]. Briefly, for each target-specific sequence, a specific RT primer was designed, located immediately downstream of the left and right hand half-probe target sequence. Following reverse transcription, left and right hand half-probes were hybridized to the cDNA at 60°C overnight. Annealed half-probes were ligated and subsequently amplified by PCR (33 cycles of 30 s at 95°C, 30 s at 58°C and 60 s at 72°C, followed by 1 cycle of 20 min at 72°C). Primers and probes were from Sigma-Aldrich Chemie (Zwijndrecht, The Netherlands) [29,63] and RT-MLPA reagents from MRC-Holland (Amsterdam, The Netherlands). PCR amplification products were 1:10 diluted in HiDi formamide containing 400HD ROX size standard and analyzed on an Applied Biosystems 3730 capillary sequencer in GeneScan mode (BaseClear, Leiden, The Netherlands).

Trace data were analyzed using the GeneMapper software package (Applied Biosystems). Signals below the threshold value for noise cutoff in GeneMapper (peak area <200) were assigned the threshold value for noise cut off. Subsequently, results from target genes were calculated relative to the average signal of GAPDH and assigned the threshold value if below 200.

### Analysis of cytokines, chemokines and cytotoxic molecules

HLA-E restricted T-cell clones (1x10e6 c/w) were cultured in 24 well plates in the absence or presence of adherent macrophages (2.5x10e5 c/w). Macrophages were generated from CD14^+^ monocytes that were isolated from HLA-A2 negative buffycoat PBMCs with CD14 MACS beads (Miltenyi) and differentiated for 6 days in the presence of 50 ng/ml M-CSF (R&D systems). Cultures were incubated with medium, specific or control peptide (10 μg/ml)(macrophages present), or macrophages infected with live BCG from fresh log culture (MOI = 10), or T-cell expander beads (Invitrogen) for 24 hours (no macrophages present). Supernatants were tested using the Human Cytokine, Chemokine and Immuno Cell Multiplex Assays and the Human CD8^+^ T-Cell Multiplex Assay (Merck Millipore, Amsterdam, the Netherlands). Analyses were performed on a Luminex200 with Bioplex software (Biorad, Veenendaal, the Netherlands).

### B-cell activation assays

B-cells were isolated from PBMC’s of HLA-A2 negative donors using CD19 MACS beads (Miltenyi). Purified CD19^+^ cells were plated in a 96 well round-bottom plate (5x10e4 c/w) and cultured with 5x10e4 HLA-E restricted CD8^+^ T-cells in AIMV. Also recombinant human cytokine controls for activation of B-cells in the absence of T-cells were performed (10 ng/ml of IL-4, IL-5, IL-10 and or IL-13 was used (all Peprotech)) as were the positive controls CpG (5 μg/mL CpG ODN2006 (Life Technologies)) and αIgG/IgM complex (10 μg/ml, Jackson ImmunoResearch Laboratories inc., Suffolk, UK). After 48 hours supernatants were harvested for determination of IL-6 levels by standard IL-6 ELISA (Biosource/Invitrogen) according to the manufacturer’s protocol. Cells were harvested and activation was assessed by flow cytometry with surface staining for CD80, CD86, CD40, CD25 and HLA-DR. Cells were also stained for CD3 and CD8 to exclude T-cells from the analyses (CD3-PE-TexasRed, CD19-PacificBlue (both Invitrogen), CD8-HorizonV500, CD80-PE-Cy7, CD86-FITC, CD40-APC, CD20-APC-H7 and HLA-DR-PE-Cy5 (all BD biosciences). To investigate the specificity of B-cell activation by our HLA-E restricted Mtb specific T-cell clones we performed a similar co-culture assay using well-characterized classical CD4^+^ T-cell clones generated previously. Mycobacterium specific CD4^+^ T-cell clones: R2F10 (reactive with Mtb hsp 65, HLA-DR2 restricted) produces Th1/2 cytokines, R2G7 (reactive with Mtb hsp10, HLA-DR2 restricted) Th17 cytokine profile [31]; Rp15 1-1 (reactive with Mtb hsp65, HLA-DR3 restricted) [64] Th1 cytokine profile; D2C4 (reactive with Mtb 14–22 kD fraction, HLA-DQ restricted) Th1 cytokine profile [65]. In addition to mycobacterium specific clones, we also included a Th1 CD4^+^ T-cell clone HA1.7 which recognizes influenza hemagglutinin in HLA-DR1 [66] and a cytolytic CD8^+^ T-cell clone HY21.17 recognizing the male HY antigen presented by HLA-A2 [23,67].

To address which T-cell cytokines were critical for B-cell activation, antibodies or isotype controls to block secreted cytokines were added to the B cell cultures at 20 μg/ml 2 hours prior to addition of the T-cell clones (αIL-5 (clone 14611) and αIL-13 (clone 31606)(R&D Systems), αIL-4 (clone MP4-25D2), αIL-10 (cloneJES3-19F1), isotype controls rat/ mouse IgG1 and rat IgG2a (BD Biosciences and R&D systems)) and after 48 hours supernatants were collected for determination of IL-6 production and cells were stained as described above.

### TB patients, TM staining and cytokine detection

Peripheral blood was obtained from 19 adults with TB disease (12 men, 7 women, age range 42–61 years) from the Dipartimento di Medicina Clinica e delle Patologie Emergenti, University Hospital, Palermo. TB-infected patients had clinical and radiological findings consistent with active pulmonary TB (American Thoracic Society, 2000). Diagnosis was confirmed by bacteriological isolation of *M*. *tuberculosis* in 11 patients. Other patients were classified as having highly probable pulmonary TB on the basis of clinical and radiological features that were highly suggestive of TB and unlikely to be caused by any other disease; the decision was made by the attending physician to initiate anti-TB chemotherapy, which resulted in an appropriate response to therapy. All patients were treated in accordance with Italian guidelines and received therapy for 6 months. Treatment was successful in all participants all of whom completed the full course of anti-TB chemotherapy, as shown by the absence of any clinical or radiographic evidence of recurrent disease and sterile mycobacterial cultures. Peripheral blood was collected before chemotherapy. None of the TB patients had been vaccinated with Bacillus Calmette-Guerin (BCG), or was being treated with steroid or other immunosuppressive or anti-tubercular drugs at the time of their first sampling. Three patients had evidence of HIV infection. Tuberculin (PPD) skin tests were considered positive when the induration diameter was larger than 10 mm at 72 hrs since injection of 5 U of PPD (Statens Seruminstitut, Copenhagen, Denmark). The study was approved by the Ethical Committee of the University Hospital, Palermo, where the patients were recruited. The study was performed in accordance to the principles of the Helsinki declaration and those of the “Good Clinical Practices”, and all individuals gave written informed consent to participate.

Tetramer staining was carried out as described in detail previously [68,69]. PBMC (10^6^/mL) were incubated in U-bottom 96-well plates, washed twice in phosphate buffered saline (PBS) containing 1% fetal calf serum (FCS, Sigma) and stained for 30 min at 4°C with PE-labelled tetramers (5μL each) prepared as previously described [68–70], washed and subsequently stained with FITC-labelled anti-CD8 mAb (RPA-TB, BD Biosciences) and analyzed by flow cytometry on a FACSCanto. Data were analyzed with the use of FACSDiva (BD Biosciences). Viable lymphocytes were gated by forward and side scatter and the analysis was performed on 100,000 acquired CD8 events for each sample. A cut-off of 0.01% was used as described previously [71]; values below this were set to zero.

Cytokine production following peptide stimulation was analysed by intracellular staining and flow cytometry [71]. PBMCs (10^6^/mL) were stimulated with peptides for 16 hours, the last 12 hours in the presence of monensin at 37°C in 5% CO2. The cells were harvested, washed and stained with anti-CD8 mAb (RPA-TB, BD Biosciences) in incubation buffer (PBS-1% FCS-0.1% Na azide) for 30 min at 4°C. The cells were washed twice in PBS-1% FCS and fixed with PBS-4% paraformaldehyde overnight at 4°C. Fixation was followed by permeabilization with PBS-1% FCS-0.3% saponin-0.1% Na azide for 15 min at 4°C. Staining of intracellular cytokines was performed by incubation of fixed permeabilized cells with anti-IFN-γ (25723.11, BD Biosciences), anti-IL-2 (MQ1-17H12, BD Biosciences), anti-TNF-α (MAb11, BD Biosciences), anti-IL10 (BT-10, eBioscience), anti-IL-17A (eBio64DEC17, eBioscience), anti-IL-4 (BD Biosciences, 3010.211), anti-IL13 (Biolegend, JES10-5A2) mAbs or isotypematched control mAbs, all from BD Bioscience. Cells were acquired and analyzed by FACS as described above. Analysis was performed on a minimum of 100,000 acquired CD8^+^ events for each sample.

Negative controls were background staining obtained with medium, in the absence of any stimulant. Cut-off values for a positive response were predetermined to be in excess of 0.01% responsive cells. Results below this value were considered negative and set to zero. Groups were compared using Mann-Whitney U test and p<0.05 was considered significant.

## Supporting Information

S1 Fig Flow cytometry—Gating strategy. A. Fresh human PBMCs were stained with the same antibody panels as our clones and used to determine the gating criteria for phenotyping. Top row indicates gating strategy and order of gates, subsequent analyses were performed on the CD3^+^CD8^+^ T-cells. Gates/ quadrants were set on PBMCs and applied to T-cell clones as indicated for all marker combinations. B. Example of gates for phenotyping applied to a T-cell clone.
(EPS)

S2 Fig Overview of functional profiles of T cell clones. Overall information on Th2 cytokine profile, cytolytic activity, Mtb inhibitory activity and suppressive activity of all clones tested was compiled into a summary Figure, derived from S1 Table and Figs 2C, 4A, B and C. Grey boxes indicate positive scores for cytokine production in either Luminex assay, intracellular cytokine staining or both; cytotoxic responses as measured by specific lysis of M. bovis BCG infected human macrophages; Mtb growth inhibition of infected macrophages; and the percentage of suppression of an unrelated Th1 reporter clone, according to the arbitrary cut offs shown in the legend in the figure. Blank boxes represent absence of functional responses. Nt = not tested.
(TIF)

S1 Table Raw data for all parameters analysed. Flow cytometry data are expressed as % of CD3^+^CD8^+^ cells. Cytokine data from multiplex bead arrays (‘luminex’) are secreted cytokines expressed as pg/ml, min indicates cytokine production in the absence of stimulation (macrophages present in well), max indicates cytokine production in response to αCD3/28 T-cell activator beads (no macrophages present). DcRT-MLPA RNA expression data are peak areas normalized for GAPDH expression levels.
(XLSX)
