# Supplementary figures and images for: Human CD8+ T-cells Recognizing Peptides from Mycobacterium tuberculosis (Mtb) Presented by HLA-E Have an Unorthodox Th2-like, Multifunctional, Mtb Inhibitory Phenotype and Represent a Novel Human T-cell Subset
Source: PLoS Pathog. 2015 Mar 24;11(3):e1004671. doi: 10.1371/journal.ppat.1004671 (PMC4372528; doi:10.1371/journal.ppat.1004671)

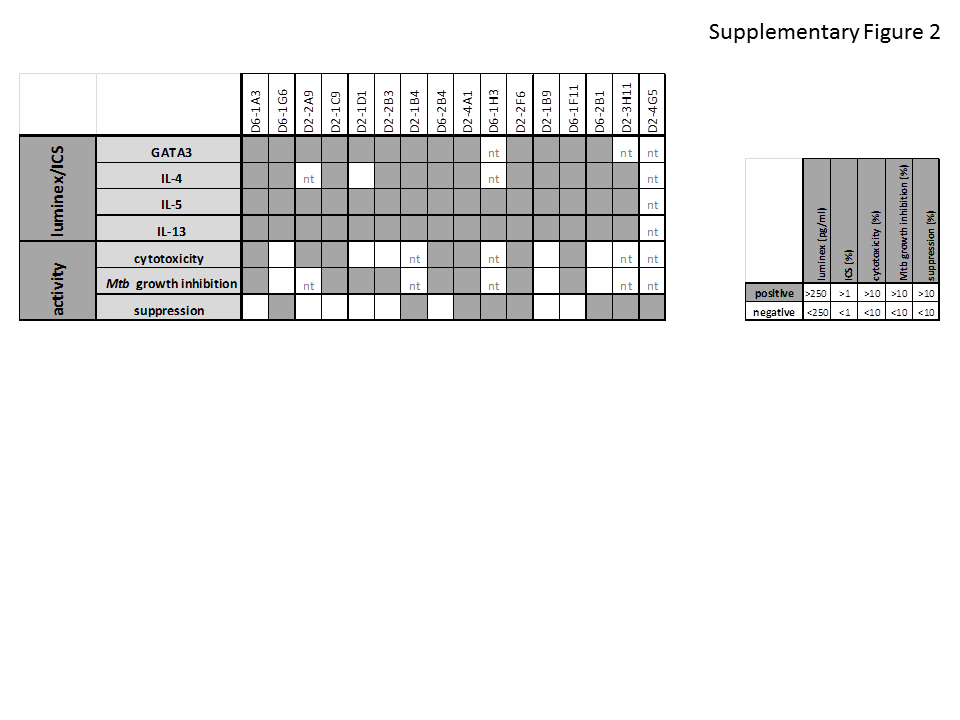

Supplement: S2 Fig — Overall information on Th2 cytokine profile, cytolytic activity, Mtb inhibitory activity and suppressive activity of all clones tested was compiled into a summary Figure, derived from S1 Table and Figs 2C, 4A, B and C. Grey boxes indicate positive scores for cytokine production in either Luminex assay, intracellular cytokine staining or both; cytotoxic responses as measured by specific lysis of M. bovis BCG infected human macrophages; Mtb growth inhibition of infected macrophages; and the percentage of suppression of an unrelated Th1 reporter clone, according to the arbitrary cut offs shown in the legend in the figure. Blank boxes represent absence of functional responses. Nt = not tested. (TIF) [file ppat.1004671.s002.tif]
